# Bmi1 Enhances Tumorigenicity and Cancer Stem Cell Function in Pancreatic Adenocarcinoma

**DOI:** 10.1371/journal.pone.0055820

**Published:** 2013-02-20

**Authors:** Erica Proctor, Meghna Waghray, Cheong Jun Lee, David G. Heidt, Malica Yalamanchili, Chenwei Li, Filip Bednar, Diane M. Simeone

**Affiliations:** 1 Department of Surgery, University of Michigan Medical School, Ann Arbor, Michigan, United States of America; 2 Department of Molecular and Integrative Physiology, and University of Michigan Medical School, Ann Arbor, Michigan, United States of America; 3 Translational Oncology Program, University of Michigan Medical School, Ann Arbor, Michigan, United States of America; University of Saarland Medical School, Germany

## Abstract

**Background:**

Bmi1 is an integral component of the Polycomb Repressive Complex 1 (PRC1) and is involved in the pathogenesis of multiple cancers. It also plays a key role in the functioning of endogenous stem cells and cancer stem cells. Previous work implicated a role for cancer stem cells in the pathogenesis of pancreatic cancer. We hypothesized that Bmi1 plays an integral role in enhancing pancreatic tumorigenicity and the function of cancer stem cells in pancreatic ductal adenocarcinoma.

**Methods:**

We measured endogenous Bmi1 levels in primary human pancreatic ductal adenocarcinomas, pancreatic intraepithelial neoplasias (PanINs) and normal pancreas by immunohistochemistry and Western blotting. The function of Bmi1 in pancreatic cancer was assessed by alteration of Bmi1 expression in several cell model systems by measuring cell proliferation, cell apoptosis, in vitro invasion, chemotherapy resistance, and in vivo growth and metastasis in an orthotopic model of pancreatic cancer. We also assessed the cancer stem cell frequency, tumorsphere formation, and in vivo growth of human pancreatic cancer xenografts after Bmi1 silencing.

**Results:**

Bmi1 was overexpressed in human PanINs, pancreatic cancers, and in several pancreatic cancer cell lines. Overexpression of Bmi1 in MiaPaCa2 cells resulted in increased proliferation, in vitro invasion, larger in vivo tumors, more metastases, and gemcitabine resistance while opposite results were seen when Bmi1 was silenced in Panc-1 cells. Bmi1 was overexpressed in the cancer stem cell compartment of primary human pancreatic cancer xenografts. Pancreatic tumorspheres also demonstrated high levels of Bmi1. Silencing of Bmi1 inhibited secondary and tertiary tumorsphere formation, decreased primary pancreatic xenograft growth, and lowered the proportion of cancer stem cells in the xenograft tissue.

**Conclusions:**

Our results implicate Bmi1 in the invasiveness and growth of pancreatic cancer and demonstrate its key role in the regulation of pancreatic cancer stem cells.

## Introduction

Pancreatic ductal adenocarcinoma (PDA) is a highly aggressive epithelial cancer with the worst prognosis of any major malignancy with a reported 5-year survival rate of approximately 5%. It is the fourth leading cause of cancer death per year in the United States and eighth worldwide with an expected incidence of 43,920 cases in 2012 in the United States alone [1]. Despite advances in our understanding of this disease, the molecular events underlying the development and progression of pancreatic cancer are still largely unknown and may hold the key to the development of more efficacious and novel therapeutic strategies.

B-cell-specific Moloney murine leukemia virus insertion site 1 (Bmi1) is a member of the Polycomb group family of proteins that was initially found to induce murine lymphoma formation upon cooperation with c-Myc [2], [3]. The oncogenic modulation of Bmi1 has been further elucidated in several aspects of cell proliferation and development. Bmi1 has been shown to play a critical role in cell cycle regulation by acting as a transcriptional repressor of the INK4a/ARF locus [4], [5]. Dysregulation by Bmi1 via stable inactivation of the p16INK4a-pRb and the p14ARF-MDM2-p53 pathways is implicated in the oncogenesis of the hematopoietic system [6], [7] and in the development of small cell carcinoma in the lung [8]. Bmi1 also has the capacity to target other aspects of cell senescence, as overexpression of Bmi1 has been shown to immortalize normal fibroblasts and mammary epithelial cells via reactivation of the human telomerase reverse transcriptase gene in these cells [9]. Additionally, robust evidence suggests that Bmi1 is critical to the invasive potential and contributes to tumorigenic capacity in colon cancer [10], medulloblastoma [11], laryngeal cancer [12], breast cancer [13], and prostate cancer [14]. Recent studies also implicate Bmi1 as a crucial protein for the maintenance and self-renewal of normal stem cells, including hematopoietic, neural, myeloid and squamous stem cells [15], [16], [17], [18] as well as cancer stem cells in several tumor types [14], [19], [20], [21]. Bmi1 has been found to sustain cancer stem cell renewal in glioblastoma multiforme and to determine the proliferative capacity of leukemic stem cells [22], [23]. Moreover, loss of Bmi1 has been observed to prevent the progression of lung tumors in an oncogenic K-ras-initiated mouse model of lung cancer through inhibition of bronchiolalveolar stem cells [24].

Bmi1 has been recently implicated in several aspects of pancreatic biology. Regulation of the INK4a locus by Bmi1 and MLL1 has been implicated in the maintenance of pancreatic β cell proliferation and the capacity of β cells to recover after pancreatic islet damage [25]. Bmi1 expressing acinar and islet cells have been found in the murine pancreas and Bmi1 plays a key role in the recovery of the acinar compartment after cerulein-induced pancreatitis and diphtheria toxin-mediated acinar cell ablation in mice [26], [27]. Overexpression of Bmi1 has been noted in human pancreatic cancer samples compared to the normal pancreas [28], [29], [30]. Bmi1 was upregulated in pancreatic tumors arising in the Ela-tTa, TetO-Cre, KrasG12V genetically engineered mouse model of pancreatic cancer [28]. Similar observations were made during cerulein-induced pancreatitis [28]. Overexpression of Bmi1 has been correlated with worse prognosis in a small cohort of pancreatic cancer patients [29]. While these data potentially implicate Bmi1 in pancreatic tumorigenesis, we have very limited understanding of the underlying mechanisms of Bmi1 function. In this study, we examine the functional significance of Bmi1 expression in pancreatic adenocarcinoma using primary human xenograft models and pancreatic cancer cell lines. Our work reveals that Bmi1 supports human pancreatic cancer growth by regulating cell cycle progression and maintaining the pancreatic cancer stem cell compartment.

## Results

### Bmi1 is highly expressed in PanIN lesions, pancreatic adenocarcinomas, and in select pancreatic cancer cell lines

**Figure 1:**
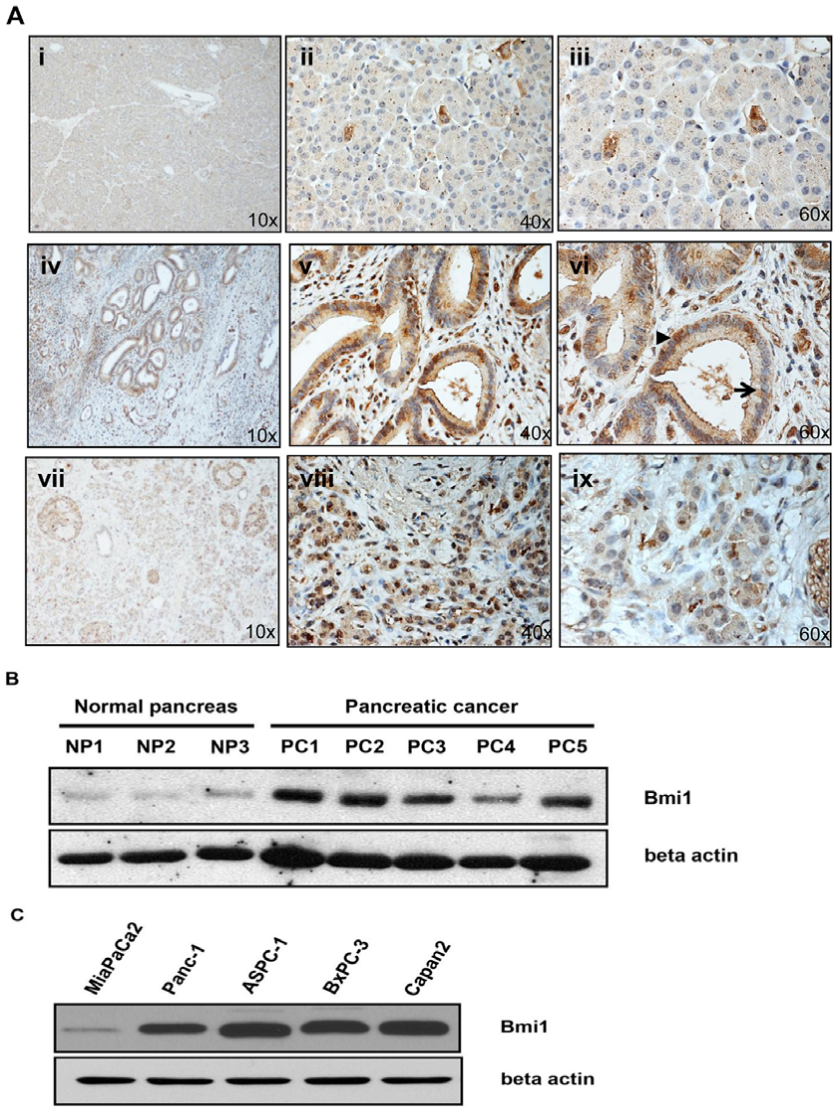
Bmi1 is overexpressed in PanIN lesions, pancreatic adenocarcinomas, and pancreatic cancer cell lines. **A**. Immunohistochemistry for Bmi1 expression was performed on pancreas tissue specimens with varying degrees of dysplasia ranging from normal (n = 10) through PanIN (n = 16) to invasive adenocarcinoma of the pancreas (n = 10). As negative controls, we subjected tissue specimens to the staining procedure in the absence of specific Bmi1 antibody (1∶100, Cell signaling). The images were captured at 10x, 40x, and 60x magnification. Representative images show few cells expressing Bmi1in normal pancreas tissue (i, ii, iii), high levels of expression in PanIN lesions (iv, v, vi), and significant overexpression in adenocarcinoma (vii, viii, ix). **B**. Western blot analysis of Bmi1 expression in normal pancreas lysates (n = 10) and pancreatic cancer tissue lysates (n = 10) from different patients was performed. Bmi1 is overexpressed in tumor lysates compared to normal pancreas tissue controls. Beta actin was used as the loading control. **C**. Endogenous Bmi1 expression in pancreatic cancer cell lines was determined by Western blot analysis. β-actin served as a loading control.

We first sought to determine the expression of Bmi1 in samples of human primary pancreatic cancers and compared expression levels to samples of normal pancreas. Tissue sections from 10 different primary human pancreatic adenocarcinomas, 10 normal pancreas samples, and 16 preneoplastic PanIN lesions with varying degrees of epithelial dysplasia (PanIN 1 to PanIN3) were examined for Bmi1 expression following immunohistochemical staining. Upon microscopic evaluation, we found that PanIN lesions and sections of human pancreatic adenocarcinoma showed marked upregulation of expression of Bmi1 when compared to normal pancreas tissues (Figure 1A). A significant number of PanIN lesions (with moderate to severe dysplasia) and adenocarcinoma sections stained for both cytoplasmic and nuclear Bmi1 (arrows in Figure 1A panel vi). Interestingly, Bmi1 staining was predominately expressed in the dysplastic glandular tissue of both PanIN and adenocarcinoma samples, with much less pronounced staining detected in a subset of cells in the stromal component of the neoplastic samples. In total, Bmi1 was expressed in 10/16 PanIN lesion sections and in 8/10 primary pancreatic adenocarcinomas. RT-PCR (data not shown) and Western blot analysis of normal and cancer tissues (Figure 1B) recapitulated the findings seen in our immunohistochemical analysis. We also examined five pancreatic cancer cell lines, MiaPaCa2, Panc-1, AsPC-1, BxPC-3 and Capan2 for the expression of Bmi1 using Western blot analysis (Figure 1C). High levels of Bmi1 expression were noted in BxPC3, Panc-1, AsPC-1, and Capan2 human pancreatic cancer cell lines, while expression was low in the MiaPaCa2 pancreatic cancer cell line. These data indicate that Bmi1 expression is common in neoplastic pancreatic tissue and in pancreatic cancer cell lines. The high levels of Bmi1 expression seen in precursor lesions of pancreatic cancer (PanINs) also suggest that expression of Bmi1 occurs at an early stage in pancreatic oncogenesis and may potentially play a role in pancreatic cancer progression.

### Bmi1 affects pancreatic cancer cell proliferation in vitro

**Figure 2:**
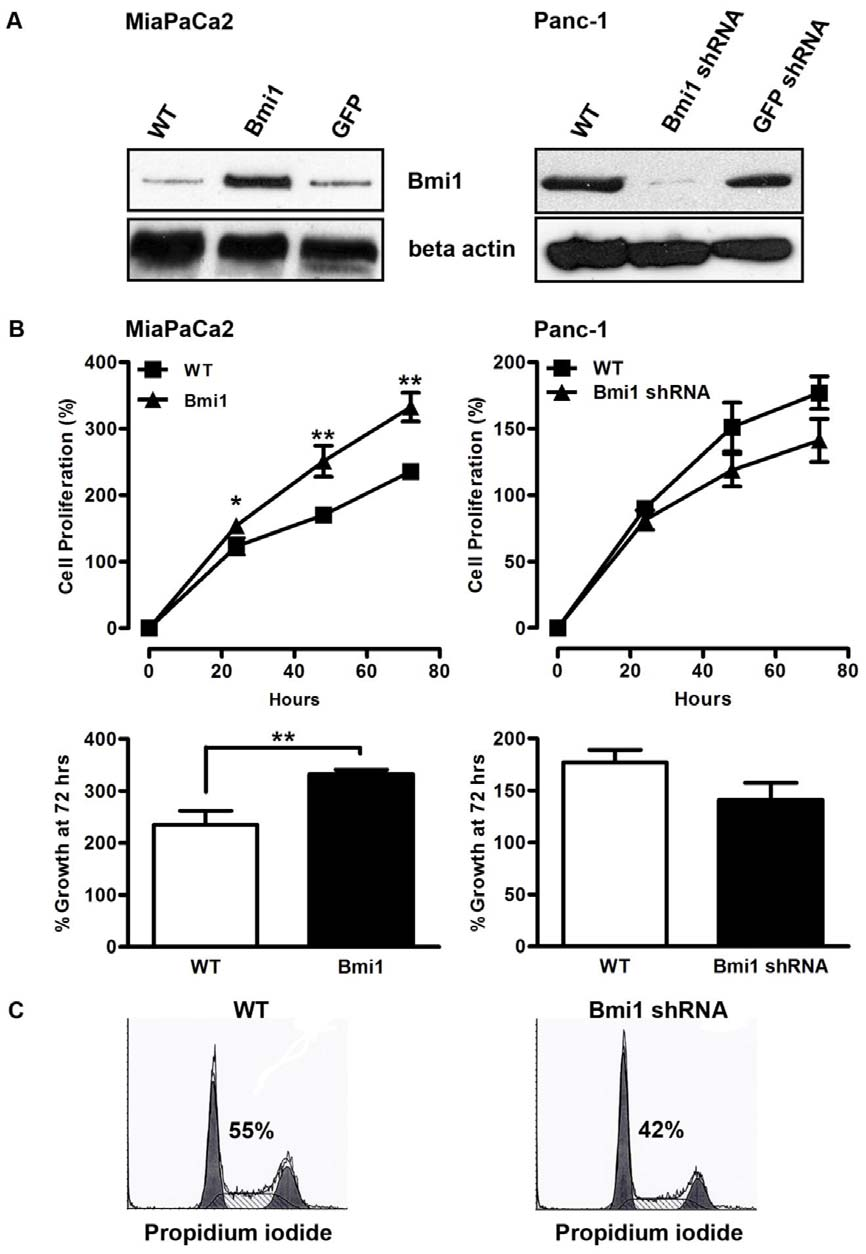
Altered Bmi1 expression affects cell proliferation *in vitro*. **A**. Bmi1 protein expression was analyzed following transfection of MiaPaCa2 (top) and Panc-1 (bottom) pancreatic cancer cells with a lentiviral vector (Bmi1) encoding Bmi1 or Bmi1 shRNA (Bmi1 shRNA) as described previously [40]. A lentiviral vector expressing GFP (GFP) or control shRNA was used as negative control. **B.** *In vitro* cell proliferation of MiaPaCa2 cells (left) and Panc-1 cells (right) following modulation of Bmi1 expression was assessed by the MTS assay (Promega, Madison, WI). Cell replication was recorded as calculated percent proliferation increase derived from reagent uptake at time (t) minus basal reagent uptake at day 0. Bottom panels show the observed changes in proliferation 72 hours after plating. Data are expressed as the mean ± SEM. * p<0.05, ** p<0.0001, n = 6 independent experiments. **C**. Representative cell cycle histograms following flow cytometric analysis of propidium iodide (PI) stained Panc-1 cells show reduction in the percentage of cells entering S-phase upon Bmi1 expression inhibition.

Given the increased level of expression of Bmi1 in pancreatic epithelial neoplastic tissue and its known cell cycle regulatory role, we next asked if its overexpression plays a functional role in pancreatic cancer cells. We chose the Panc-1 and MiPaCa2 cell lines for these experiments due to their differential levels of Bmi1 expression (Figure 1C). We overexpressed Bmi1 in MiaPaCa2 cells and silenced Bmi1 in Panc-1 cells with lentiviral constructs. Overexpression or knockdown of Bmi1 in these cell lines was confirmed by Western blot analysis (Figure 2A). Transfection of control lentiviral-GFP vector alone did not affect Bmi1 expression in either cell line (Figure 2A). Increased expression of Bmi1 in MiaPaCa2 cells significantly increased cell proliferation compared to control MiaPaCa2 cells (235±11% vs. 333±9% at 72 hours, p<0.0001, n = 6 experiments, Figure 2B). Silencing of Bmi1 expression in the Panc-1 cell line inhibited cell proliferation compared to lentiviral control shRNA infected cells, although our results did not reach statistical significance (177±12% vs. 141±16% at day 4, p = 0.11, n = 6 experiments, Figure 2B). These data show that increased levels of expression of Bmi1 in pancreatic cancer cell lines enhances cellular proliferation.

### Changes in Bmi1 expression result in altered progression through the cell cycle but no changes in the rate of apoptosis

The observed changes in cell proliferation after Bmi1 expression changes could result from changes in cell cycle progression with or without a change in the rate of apoptosis. We used flow cytometric analysis with propidium iodide to study the distribution of the Panc-1 and MiaPaCa2 cell lines with altered levels of Bmi-1 in the various phases of the cell cycle to address this point. In MiaPaCa2 cells, induction of Bmi1 expression showed a mild but non-significant increase in the percentage of cells entering the S-phase (44.7±1.7% to 50.7%±2.3%, p = 0.63, n = 3 experiments, data not shown). The change of Bmi1 expression led to a more pronounced effect in Panc-1 cells, as the loss of Bmi1 in these cells caused a significant decrease in the percentage of actively dividing S-phase cells from 54.9±2.5% to 42.0±1.4% (*p<0.05, n = 3 experiments, Figure 2C). Alteration in the expression of Bmi1 did not cause significant changes in apoptosis as measured by the sub-G0/G1 fraction and TUNEL staining in both MiaPaCa2 and Panc-1 cell lines (data not shown).

### Bmi1 enhances pancreatic cancer cell invasion capacity

**Figure 3:**
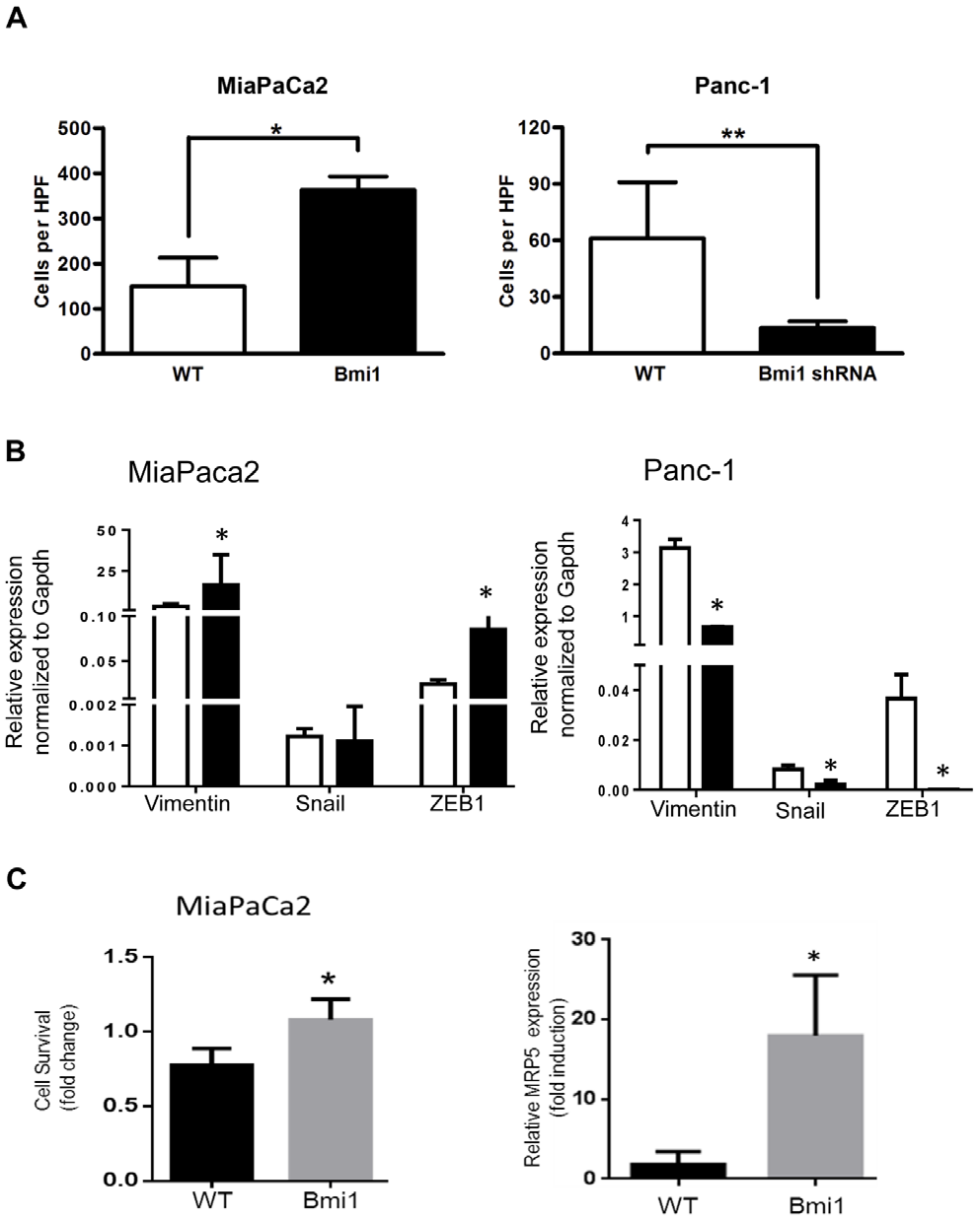
Bmi1 expression alters *in vitro* pancreatic cancer cell invasion and induces a chemoresistant phenotype. A. Overexpression of Bmi1 in MiaPaCa2 cells enhances their invasiveness in in vitro Matrigel invasion assays (left panel). In contrast, Bmi1 downregulation in Panc-1 cells leads to decreased tumor cell invasion (right panel). Data are expressed as the mean ± SEM. * p<0.0003, ** p<0.004, n = 6 independent experiments. B. Overexpression of Bmi1 in MiaPaCa2 cells leads to upregulation of the EMT markers vimentin, and ZEB1, while downregulation of Bmi1 in Panc-1 cells leads to reduced expression of EMT markers. Data are expressed as the mean± SEM compared to control cells (* p<0.005). C. Cell survival of GFP or Bmi1 transfected MiaPaCa2 cells (left) following treatment with gemcitabine for 48 hrs. Data was recorded as fold change over control untreated group and expressed as the mean ± SEM (* p<0.05 vs control). q-RT-PCR analysis of MRP5 expression in GFP or Bmi1 transfected MiaPaCa2 cells (right) following gemcitabine treatment. Data are normalized to GAPDH and expressed as the mean ± SEM (* p<0.05 vs control).

Bmi1 overexpression has previously been associated with poorer prognosis and a more invasive phenotype in other malignancies. We asked if Bmi1 expression had such an effect in pancreatic cancer cells using the Matrigel in vitro invasion assay. MiaPaCa2 cells with elevated Bmi1 expression showed nearly 2-fold increased capacity (149±26 vs 363±30, *p<0.0003) for invasion when compared to wild type control vector cells (Figure 3A, left). Silencing of Bmi1 in Panc-1 cells resulted in a markedly reduced capacity for the cells to undergo invasion (61±12 vs 14±4, p<0.004, Figure 3A, right). These data suggest that Bmi1 expression not only enhances proliferation but also the invasive capacity of pancreatic cancer cells. Since Bmi1 expression enhanced cellular invasion, we tested the ability of Bmi1 to modulate regulators of epithelial mesenchymal transition (EMT), a process which is associated with an invasive phenotype. We found increased Bmi1 expression in MiaPaCa-2 cells did correlate with increased expression of the EMT markers vimentin and ZEB1, while knockdown of Bmi1 in Panc-1 cells lead to downregulation of these markers and Snail (Figure 3B), demonstrating a clear relationship between Bmi1 and expression of EMT markers in pancreatic cancer cells.

### Bmi1 enhances gemcitabine resistance

The EMT phenotype has been described to contribute to not only pancreatic cancer invasion but also treatment resistance in pancreatic cancer cells [31], [32]. We therefore hypothesized that Bmi-1 might regulate resistance against chemotherapeutic agents in pancreatic cancer cells. To test this hypothesis, MiaPaCa2 cells expressing GFP alone or Bmi1 were left untreated or treated with 100 µM gemcitabine for 48 hrs and assays were performed to assess cell survival. Quantitative RT-PCR was performed to measure changes in expression of ABC transporter gene MRP5. Members of MRP family are important in mediating drug resistance, and MRP5 has been shown to be expressed at significantly high levels in pancreatic cancer compared to control pancreatic tissues, suggesting it might have a role in drug resistance [33]. MiaPaCa2 cells with elevated expression of Bmi1 had increased resistance to cell death in response to gemcitabine and increased expression of the drug resistance gene MRP5 compared to control cells. These data suggest Bmi1 participates in therapeutic resistance in pancreatic cancer cells and that MRP5 may contribute to this phenotype.

### Bmi1 enhances pancreatic cancer cell tumorigenicity in vivo

**Figure 4:**
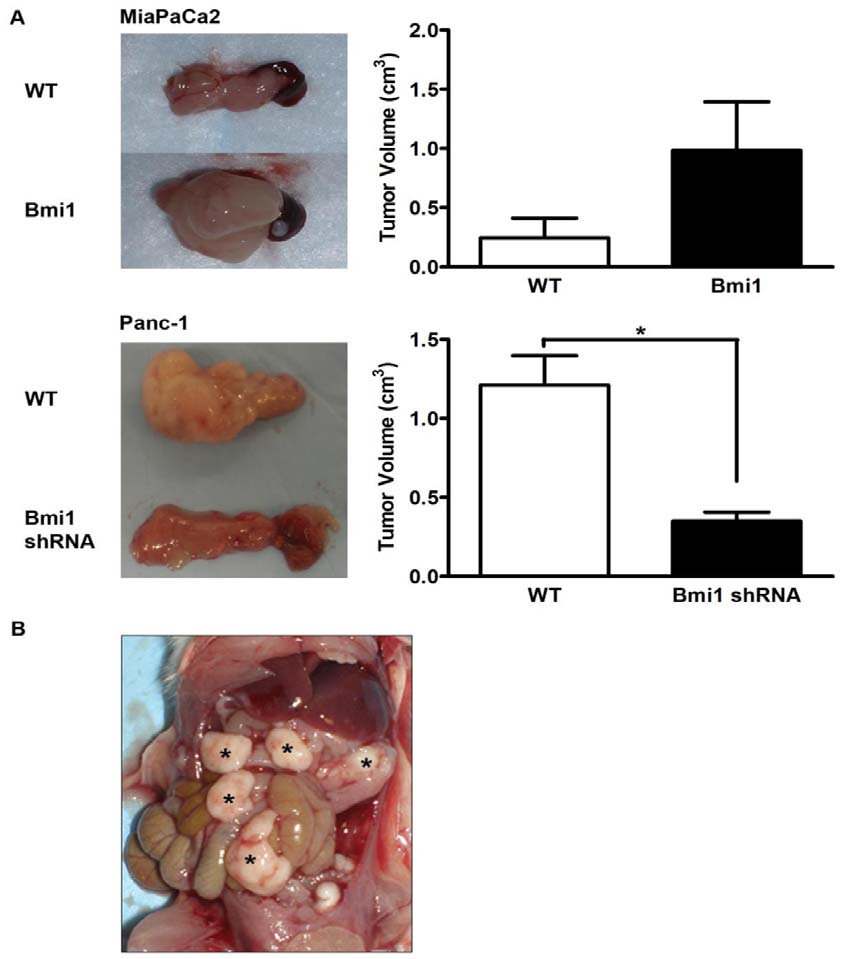
Modulation of Bmi1 expression affects tumor growth and invasion *in vivo*. **A**. Representative images shown demonstrate relative tumor size difference at 28 days following orthotopic pancreas implantation of MiaPaCa2 (top) and Panc-1 cells (bottom) in NOD-SCID mice after modulating Bmi1 expression. The tumor volume was calculated by using the formula for volume of an ellipsoid structure (4/3 * π * width/2 * length/2 * height/2). Data are expressed as the mean ± SEM (* p<0.05, n = 5 independent experiments). **B**. The image shown is representative of enhanced metastatic potential in MiaPaCa2 cells following induced expression of Bmi1. * Marked sites show gross metastasis of MiaPaCa2 tumor to extrapancreatic sites in NOD-SCID mice 28 days following orthotopic injection of cells.

Since our in vitro studies suggested that Bmi1 plays a regulatory role in pancreatic cancer cell proliferation and invasion, we wanted to address the biological significance of these results in an in vivo orthotopic model of pancreatic cancer. To address this point, we injected lentiviral transduced cell lines into the pancreatic tails of recipient NOD/SCID mice and measured the resulting tumor growth and development of extrapancreatic metastases in these mice. Following induction of Bmi1 expression, MiaPaCa2 cells showed significantly greater *in vivo* tumor growth. Bmi1 induced MiaPaCa2 cells showed enhanced engraftment, with 100% (5/5) orthotopic tumor formation while only 3/5 mice injected with wild type MiaPaCa2 cells showed pancreatic tumor engraftment (Table 1). Moreover, a trend to larger tumor size was seen in the tumors formed by Bmi1 expressing cells compared to control MiaPaCa2 cells (0.27±0.17 cm3 vs. 0.98±0.41 cm3, p  =  NS, Figure 4A). All mice injected with either control Panc-1 cells (5/5) or Bmi1 silenced Panc-1 cells (5/5) showed tumor formation in the pancreas, however Bmi1 silenced Panc-1 cells formed tumors that were 2.5 times smaller on average than tumors derived from control Bmi1 expressing Panc-1 cells (1.21±0.23 cm3 vs. 0.42±0.09 cm^3^, *p<0.05, Figure 4A, bottom panel). In addition to larger tumor size, Bmi1 expressing cells displayed enhanced metastatic potential *in vivo*. Necropsies performed in animals 28 days following injection of Bmi1 expressing MiaPaCa2 cells revealed gross metastasis of tumor to extra-pancreatic sites, including the peritoneum, liver and bowel in (3/5 animals, Figure 4B), while no gross metastatic deposits (0/5) observed in mice injected with wild type MiaPaCa2 cells at 28 days (Table 1). These data corroborate our in vitro observations and support the notion that Bmi1 coordinates the proliferative and invasive programs in pancreatic cancer cells.

**Table 1:** The effect of BMi-1 on tumor engraftment, growth and metastasis.

	Engraftment	Metastases	Tumor size (cm^3^)
Panc-1	5/5	0/5	1.21±0.23
Panc-1 + Bmi1 shRNA	5/5	0/5	0.42±0.09
MiaPaCa2	3/5	0/5	0.27±0.17
MiaPaCa2 + Bmi1	5/5	3/5	0.98±0.41

### Bmi1 is highly expressed in pancreatic cancer stem cells and controls their self-renewal

**Figure 5:**
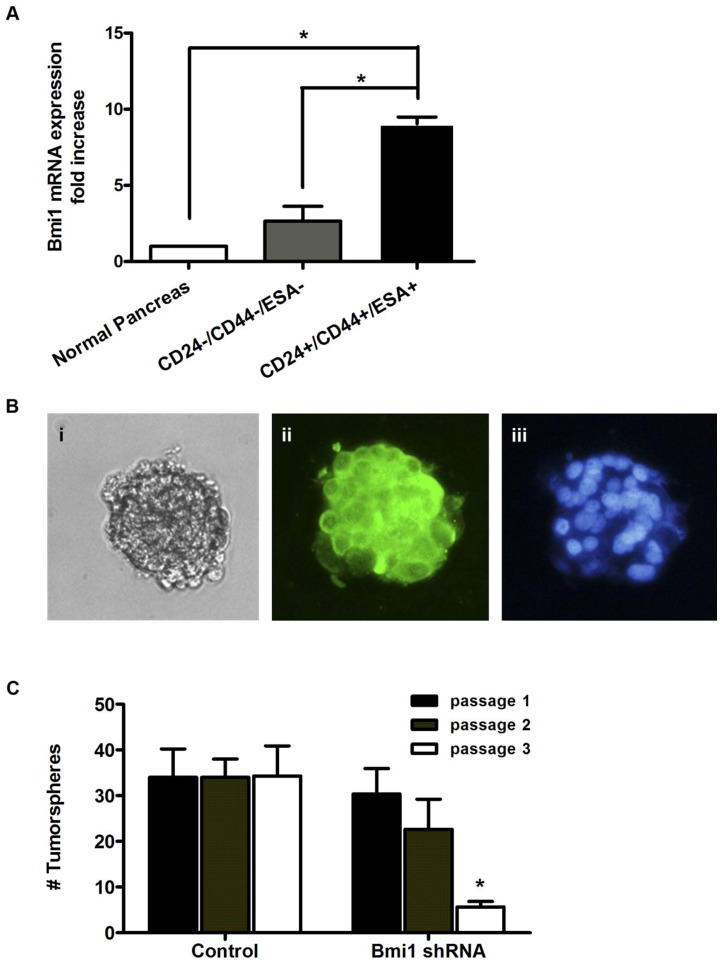
Bmi1 is overexpressed in pancreatic cancer stem cells and regulates their self-renewal. **A.** CD44^+^/CD24^+^/ESA^+^ cells were isolated using flow cytometry from pancreatic cancer tissues as described previously [34]. Total RNA was isolated and mRNA was quantitated by q- RT-PCR in normal pancreas, bulk CD44^–^/CD24^–^/ESA- pancreatic cancer cells and CD44^+^/CD24^+^/ESA^+^ pancreatic cancer cells. Data are expressed as the mean ± SEM (* p<0.05, n = 3 independent experiments, each performed in triplicate). **B.** Immunofluorescent staining of Bmi1 in tumorspheres formed from CD44^+^/CD24^+^/ESA^+^ cells. **C.** Silencing Bmi1 inhibits the ability of CD44^+^/CD24^+^/ESA^+^ cells to form spheres with serial passaging. Data are expressed as the mean ± SEM (* p<0.05, n = 3 independent experiments, each performed in triplicate).

Our laboratory has previously identified a population of highly tumorigenic subpopulation of cells that displays properties of self-renewal and differentiation in pancreatic cancer [34]. We hypothesized that Bmi1 may play a functional role in these pancreatic cancer stem cells (CSCs) given its involvement in other stem cell systems. We turned to low passage primary human pancreatic cancer xenografts to address this question. Utilizing fluorescence-activated cell sorting (FACS), we isolated the CSCs through the combination of CD44, CD24, and ESA (also known as EpCAM or epithelial cell adhesion molecule) cell surface expression [34]. Quantitative RT-PCR was performed to measure Bmi1 levels in the CSCs (cell surface markers CD44^+^CD24^+^ESA^+^) and compared to the remaining bulk tumor cells harvested from the human pancreatic cancer xenografts grown in NOD-SCID mice. Normal pancreas extracts served as an additional control. Notably, pancreatic CSCs have a significantly higher expression of Bmi1 mRNA (approximately 9-fold, *p<0.05) in comparison to normal cells and marker negative bulk tumor cells (up to 3-fold, p<0.05, Figure 5A).

Sorted CSCs were also tested *in vitro* in tumorsphere assays to determine if Bmi1 participates in CSC renewal upon serial passaging. Expression of Bmi1 in tumorspheres was assessed via immunofluorescence microscopy (Figure 5B). High levels of Bmi1 expression were seen in tumorspheres enriched for cancer stem cells which corresponded to the observed elevation in Bmi1 mRNA expression levels on RT-PCR. We then transduced the tumorspheres with Bmi1 shRNA-containing or control lentivirus and serially passaged the spheres. First passage sphere formation was not significantly different between Bmi1 silenced CSCs and lentiviral infected controls (34±6 units/HPF vs. 30±6 units/HPF, p  =  NS, Figure 5C). However, upon subsequent passaging, Bmi1 silenced cells displayed decreasing capacity to form new tumorspheres. At passage three, Bmi1 silenced cells showed an approximately 7-fold decrease in their ability to form new tumorspheres when compared to wild type controls (34±7 units/HPF vs. 6±1 units/HPF, p<0.05, Figure 5C). These experiments implicate Bmi1 in the regulation of pancreatic CSC renewal.

### Bmi1 silencing in CSCs results in smaller tumors and decreased CSCs self-renewal in vivo

**Figure 6:**
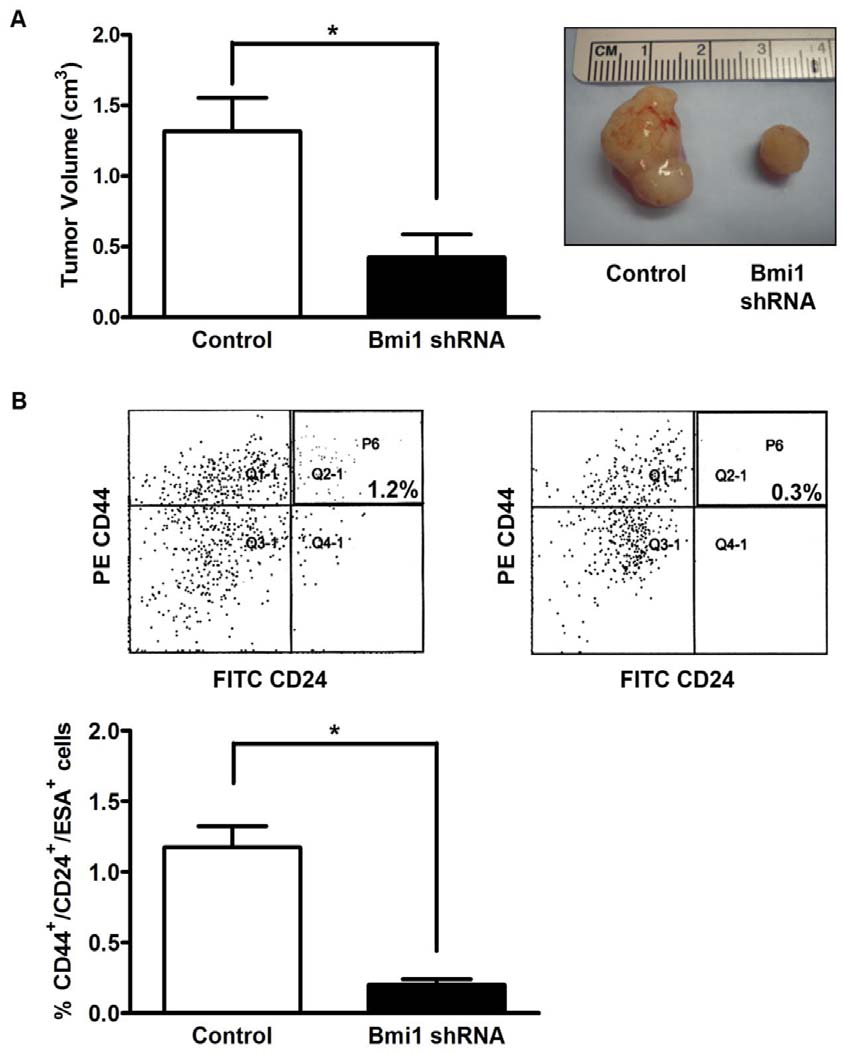
Silencing of Bmi1 decreases tumor growth and CD44^+^/CD24^+^/ESA^+^ cell numbers in tumor xenografts. **A**. Silencing of Bmi1 inhibits tumor proliferation. Representative photograph (top) of tumor xenografts shown. Left – control tumor, right – Bmi1 silenced tumor. Bar graph shows tumor volume difference at 28 days. Data are expressed as the mean ± SEM (* p<0.05, n = 4 independent experiments). **B**. Silencing of Bmi1 directly impacts CSC number in tumor xenografts. Representative flow cytometry plots (top) of CD44^+^/CD24^+^/ESA^+^ cell numbers in control (left) and Bmi1 silenced (right) xenografts at 28 days. Bar graph (bottom) quantifies CSC numbers in xenografts at 28 days following silencing of Bmi1. Data are expressed as the mean ± SEM (* p = 0.0007, n = 4 independent experiments).

To test the in vivo relevance of the tumorsphere experiments, we implanted equal numbers of Bmi1 silenced and control lentiviral transfected CD44^+^CD24^+^ESA^+^ CSCs derived from primary human pancreatic cancer xenografts in the subcutaneum of NOD/SCID mice and measured the resultant tumor growth. Bmi1 silenced tumors were significantly smaller compared to the control tumors when analyzed 30 days following implantation (0.4±0.2 cm^3^ vs. 1.3±0.2 cm^3^, *p<0.05, Figure 6A). When the tumors were analyzed for their CSC content via flow cytometry, Bmi1 silenced tumors showed a significant decrease in the CD44^+^CD24^+^ESA^+^ CSC population in comparison to their control counterparts (0.20±0.04% vs. 1.2±0.1%, * p = 0.0007, Figure 6B). This in vivo data, along with the in vitro tumorsphere results, supports the role of Bmi1 in the maintenance of the pancreatic CSC compartment by regulating their self-renewal.

## Discussion

The present study provides clear evidence for the integral involvement of Bmi1 in the pathogenesis of pancreatic cancer. Overexpression of Bmi1 was consistently observed in pancreatic cancer tissues compared with normal controls. Most human pancreatic cancer cell lines were also observed to have high endogenous expression of Bmi1. Importantly, Bmi1 overexpression was observed in a majority (10/16) of the preneoplastic PanIN lesions with moderate to severe degrees of epithelial dysplasia. This data suggests that Bmi1 expression occurs at a relatively early stage in pancreatic carcinogenesis and corroborates previously published observations in human and murine pancreatic cancer models [28], [29], [30]. Despite these results, a functional role for Bmi1 has not been previously elucidated in human or murine pancreatic cancer.

We functionally validated the importance of Bmi1 expression in pancreatic cancer using pancreatic cancer cell lines and primary human tumor xenografts. Bmi1 overexpression in MiaPaCa2 pancreatic cancer cells led to increased proliferation and enhanced invasion in in vitro Matrigel assays. Conversely Bmi1 knockdown in Panc-1 cells inhibited their proliferation. These observations were further confirmed by implantation in NOD-SCID mice, where Bmi1 overexpression in MiaPaCa2 cells resulted in increased tumor size and metastatic ability of the cells. In contrast, although Panc-1 cells had equivalent engraftment regardless of the Bmi1 status, the tumors resulting from the Panc-1 cells lacking Bmi1 were significantly smaller than those from control Panc-1 cells. Increased Bmi1 expression has been associated with increased aggression in other cancer types [6], [10], [35], [36] and correlates with tumor invasion and metastasis in breast cancer [10], [13]. Our findings in human pancreatic cancer cell lines and primary human xenografts further extend these observations and implicate Bmi1 in inducing EMT and drug resistance thereby regulating pancreatic cancer growth and aggressiveness.

Tumor initiating or cancer stem cells have been isolated in human pancreatic cancer [34]. As in normal stem cells, Bmi1 is thought to be a critical component of the machinery that maintains self-renewal in CSCs. This was demonstrated to be the case in the hematopoietic system as well as the breast, brain, and prostate [14], [17], [19], [20]. In our study, when Bmi1 expression was silenced in the CD44^+^CD24^+^ESA^+^ pancreatic CSC population, both *in vitro* CSC propagation and *in vivo* tumor growth were significantly inhibited. Furthermore, a marked decrease in the number of CSCs was noted when Bmi1 silenced tumors were analyzed for their CSC content. These data for the first time experimentally implicate Bmi1 as an important regulator of pancreatic CSC maintenance and pancreatic tumor initiation. Further study is underway of the role of Bmi1 in the complex regulatory transcriptional programs controlling the renewal and maintenance of the stem cell state. Bmi1 has been implicated in the stem cell renewal and proliferation in many normal as well as cancerous tissues including the hematopoietic system, nervous system, the breast, the prostate, lungs, the intestine, and the normal pancreas. A critical observation, when one reviews the majority of these studies, is that Bmi1 function has mainly been implicated in these organ systems using genetically engineered mouse models. There is a paucity of data that examines Bmi1 function in human tissues directly. Our study implicates Bmi1 as a direct regulator of pancreatic tumorigenesis using primary human patient-derived low-passage xenografts and pancreatic cell lines. It complements the findings of the other studies and further cements the role of Bmi1 as a key regulator of cancer stem cell renewal, which directly impacts tumor initiation and growth in vivo. It is the first study to implicate Bmi1 directly in pancreatic tumorigenesis. It is difficult to extrapolate how Bmi1 expression in pancreatic cancer stem cells relates to normal pancreatic homeostasis as the only paper addressing Bmi1 function and not just expression in the adult pancreas focuses on a murine model and not human pancreas [27]. It is notable that in that paper Bmi1 was not expressed in cells that express the normally accepted pancreatic progenitor markers such as Pdx-1, Nestin, and Sox9, but was instead present in what appeared to be fully differentiated acinar cells. Therefore it is difficult to speculate on the role of Bmi1 in normal pancreas homeostasis in the human, but our work clearly implicates Bmi1 in human pancreatic tumorigenesis.

The underlying molecular mechanisms of Bmi1 function in cancer are not completely understood. Bmi1 regulates cell cycle progression at least partly by the transcriptional regulation of the Ink4a locus, which contains the p16Ink4a and p14Arf (p16 and p19 in mice) cyclin-dependent kinase inhibitors [4], [5]. The Ink4a locus is disrupted in many pancreatic cancers and mutations in or silencing of p16/p14 expression is implicated as a key event in pancreatic cancer progression [37]. Recent studies have shown that aberrant Bmi1 expression may not necessarily be associated with downregulation of p16INK4A expression [38], [39], [40]. Prior comprehensive molecular characterizations of pancreatic cancer cell lines have shown that the vast majority have known mutations of Kras, p53, and the Ink4a locus. Relevant to this work, both Panc-1 and MiaPaCa2 cells contain homozygous deletions of the Ink4a locus. Despite the loss of the Ink4a locus, Bmi1 is able to exert pro-tumorigenic functions in both of these cell lines. We are in the process of full molecular characterization of the primary human xenograft samples used in this study, but given the preponderance of Ink4a mutations in primary human pancreatic cancers [41], we believe that a similar conclusion is likely for our primary human xenograft samples. The finding of an essential role for Bmi1 in the regulation of cell cycle, growth, and invasion in pancreatic cancer as demonstrated in this study supports the possibility that Bmi1 regulates proliferative capacity in an INK4A-independent manner in pancreatic cancer. Given the ubiquitous nature of Bmi1 as part of PRC1, it is likely that transcriptional regulation of other genomic loci by PRC1 may be responsible for the functional effects seen during Bmi1 modulation. It will be important to more comprehensively address how epigenetic changes promote pancreatic tumorigenesis and what the relevant molecular targets of these chromatin changes are that promote cellular proliferation, survival, and dissemination.

## Materials and Methods

### Patients and Specimens

Pancreatic adenocarcinoma tissue samples and pancreatic intraepithelial neoplasia (PanIN) samples were obtained from patients who underwent pancreatic resection at the Department of Surgery, University of Michigan Medical Center from 2000–2009 using IRB-approved guidelines. The pancreatic tumor tissue and paired normal pancreatic tissue were snap-frozen and stored in liquid nitrogen until further use.

### Cell Culture

The human pancreatic cancer cell lines Panc-1, MiaPaCa2, BxPC3, AsPC-1, and BxPC-3 were grown in Dulbecco's modified Eagle's medium supplemented with 10% fetal bovine serum (Gibco/Invitrogen, California). All cell lines were maintained in a humidified atmosphere at 37°C with 5% CO_2_. For maintaining tumorspheres *in vitro*, cancer stem cell populations isolated from two different human pancreatic cancer xenografts were grown as spheroid colonies in media containing 50% NeuralBasal Media (Gibco), 1% N2 supplement (Gibco), 2% B27 supplement (Gibco), 50 µM 2-mercaptoethanol (Gibco), 1% Antibiotic-Antimycotic (Gibco), 250 mg/ml human fibronectin (Sigma), 10 ng/ml BMP4(Sigma), 10 ng/ml LIF (Sigma), 20 ng/ml human FGF-2 (Sigma) or 20 ng/ml EGF (Gibco), all in 1∶1 DMEM/F12. Cells were plated in ultralow attachment 6-well plates (Corning, New York) at a density of 25,000 cells per well. Sphere cultures were passaged every 7–10 days. To passage spheres, media was removed and spheres were incubated at room temperature for 5 minutes in 0.05% trypsin (Gibco). Spheres were observed under the microscope to verify dissociation. Cells were then washed with HBSS (Gibco) and filtered through a 40 µm strainer before replating.

### Vector production and RNA interference

Lentiviral vectors containing Bmi1, Bmi1 shRNA, control shRNA and Green Fluorescent Protein (GFP) utilized in our experiments were generously provided by Dr. Max Wicha (University of Michigan, Ann Arbor) and transfection protocols were followed as described previously [20].

### Western blotting

Whole cell lysates were prepared by incubating cells in ice-cold RIPA buffer (50 mM Tris. HCl, pH 7.4, 1% NP-40, 0.5% sodium deoxycholate, 0.1% SDS, 50 mM NaF, 5 mM sodium orthovanadate, 50 mM Sodium β-Glycerophosphate, 20 mM Sodium pyrophosphate, 5 µg/ml leupeptin, 5 µg/ml pepstatin, and 0.5 mM PMSF). Lysates were then diluted in 4× SDS sample loading buffer containing 200 mmol/L Tris-Cl (pH 6.8), 40% glycerol, 8% SDS, 0.1% bromophenol blue, and 10% β-mercaptoethanol. Lysates were then boiled at 100°C for 5 min. Equal amounts of protein were resolved by SDS-PAGE and transferred to nitrocellulose membranes. A commercially available monoclonal antibody directed against Bmi1 (1∶200, Upstate Biotechnology, Lake Placid, NY) was used as the primary antibody. After analysis, the blots were stripped, washed and reprobed with β-actin antibody (Sigma, St Louis, MO) as a loading control. Images were visualized using the ECL Detection System (Amersham, Arlington Heights, IL).

### Immunofluorescence

For immunocytology, tumorspheres were cytospun using a Shandon Centrifuge (Thermo Fisher Scientific, PA) at 800 rpm for 3 minutes onto Superfrost/Plus microscope slides (Fisher Scientific, PA). Samples were then fixed in −20°C methanol for 15 min. Thereafter, samples were air-dried and stored at 4°C until cells were stained as outlined below. Sections were rinsed in PBS +0.1% Tween 20 twice for 5 minutes. Sections were then serum blocked using species appropriate secondary antibody (10% Serum in PBS) for 30 minutes to block non-specific binding of immunoglobulin. Sections were then incubated with an anti- Bmi1 antibody (Upstate Biotechnology, Lake Placid, NY) 2 hours at room temperature or overnight at 4 °C in a humid chamber. After rinsing in PBS, the secondary antibody, either fluorescein conjugated anti-mouse, fluorescein conjugated anti-rabbit, or fluorescein conjugated anti-donkey (Jackson ImmuoResearch, PA) diluted 1∶100, was added to the sections and incubated for one hour in the dark at room temperature. Following washing with PBS, sections were mounted using Vectashield (Vector Laboratories, U.K.) mounting medium with DAPI. Slides were visualized using the Olympus BX51 microscope (Olympus Inc., NY).

### Immunohistochemistry

Paraffin sections of samples of normal human pancreas, PanIN lesions, and pancreatic adenocarcinoma were deparaffinized and rehydrated in graded alcohol. Antigen retrieval was performed by submerging slides in citrate buffer (pH 6.0, Zymed/Invitrogen Ready to use Citrate Buffer #00-5001) and heated on a hotplate to 98°C. The sections were then treated with 3% hydrogen peroxidase followed by biotin blocking in a humidified chamber at room temperature using the Dako Cytomation LSAB+Kit according to manufacturer's protocol (Dako, Denmark). Samples were incubated with a monoclonal anti-Bmi1 antibody (1∶100, Cell Signaling) at room temperature for 30 min. For negative controls, non-immune mouse IgG of the same isotype or antibody dilution solution replaced the primary antibody. After washing, the tissue sections were then incubated with biotinylated anti-mouse secondary antibody, followed by incubation with streptavidin-horseradish-peroxidase complex and developed using diaminobenzidine (DAB) substrate (Dako, Carpinteria, CA). The slides were then counterstained with hematoxylin and covered with VectaMount Mounting Media (Vector Labs, Burlingame, CA). Each stained section was then evaluated by microscopy.

### Isolation of Distinct Populations of Human Pancreatic Cancer Cells

Three individual samples of human pancreatic adenocarcinomas were obtained and implanted into eight-week-old male NOD/SCID to generate xenografts as described previously [34]. Mice were monitored weekly for tumor growth. Once the tumors reached 2 cm in size, the xenografts were procured and digested with collagenase to obtain a single-cell suspension as described previously [34]. Dissociated cells were counted and transferred to a 5 mL tube, washed twice with HBSS containing 2% heat-inactivated FBS, and resuspended in HBSS with 2% FBS at concentration of 1×10^6^ per 100 µL. Antibodies including anti-CD44 allophycocyanin, anti-CD24 phycoerythrin, and anti-H2K (PharMingen, Franklin Lakes, NJ) as well as anti–ESA-FITC (Biomeda, Foster City, CA), each at a dilution of 1∶40 were used to stain cells for subsequent flow cytometric sorting as previously described [34]. In all experiments using human xenograft tissue, infiltrating mouse cells were eliminated by discarding H2K-positive (mouse histocompatibility class I) cells during flow cytometry. Dead cells were eliminated by using the viability dye DAPI. Flow cytometry and sorting was performed using a FACS Aria cell sorter (BD Immunocytometry Systems, Franklin Lakes, NJ). Side scatter and forward scatter profiles were used to eliminate cell doublets. CD44^+^CD24^+^ESA^+^ cells were collected and analyzed in subsequent experiments.

### RT-PCR analysis

Total RNA was isolated from sorted primary human pancreatic cancer cells as described previously [34]. cDNA was first synthesized using equivalent amounts of total RNA (0.5–1 µg) with random primers in a 20 µL reverse transcriptase reaction mixture (Promega, Madison, WI). Real-time quantitative RT-PCR (Taqman) primers for Bmi1 were designed and purchased from Applied Biosystems (Foster City, CA) as Assay-on-Demand Gene Expression Products. Real-time RT-PCRs were done following the manufacturer's protocol. Twenty microliters of PCR mixture contained 10 µL of 2× Taqman Universal PCR Master Mix, 1 µL of 20× working stock of expression assay mix, and 50 ng RNA converted DNA. Real-time PCR was performed using the ABI Prism 7900HT System (Applied Biosystems, Foster City, CA). Each sample was analyzed in triplicate. Fluorescence of the PCR products was detected by the same apparatus. The number of cycles required for the amplification plot to reach the threshold limit, the *C*_t_ value, was used for quantification. Ribosomal protein S6 was used as an internal control for normalization.

### Orthotopic and subcutaneous implantation of cells in NOD-SCID mice

Eight-week-old male NOD-SCID mice were anesthetized using an intraperitoneal (i.p.) injection of 100 mg/kg ketamine and 5 mg/kg xylazine. Single cells suspensions were made with serum-free RPMI/Matrigel (BD Bioscience, San Jose, CA) mixture (1∶1 volume) and injected subcutaneously into the right and left midabdominal area using a 30-gauge needle. For orthotopic implantation, a single cell suspension of 100,000 cells/100 µL was resuspended in Matrigel and subsequently injected into the tail of the pancreas using a 30-gauge needle at volumes of 50–100 µL. Mice were monitored daily for tumor growth. Animals underwent autopsy at 28 days after cell implantation and tumor growth was assessed. Harvested tissues were fixed in neutral-buffered formalin overnight at room temperature and submitted for paraffin embedding and sectioning in the Histopathology Core laboratory. Histological analysis of the tissues was performed as detailed above.

### Cell proliferation assays

Cells were cultured at a density of 3×10^3^ cells per well in 96-well plates in DMEM media with 10% FBS. After 24 hours, CellTiter 96® Aqueous One Solution Reagent (Promega, Madison, WI) was added to each well according to the manufacturer's instructions. After 2 hours in culture, cell viability was determined by measuring the absorbance at 490 nm using a 550 BioRad plate-reader (Bio-Rad, Hertfordshire, UK).

### Apoptosis assays

TUNEL analysis and cell cycle analysis via flow cytometry were used to detect apoptosis. For the TUNEL assay, cultured MiaPaCa2 and Panc-1 cells or sectioned and fixed xenograft tumors of MiaPaCa2 and Panc-1 cells were assessed for the presence of apoptotic cells using the Promega TUNEL assay kit (Promega, San Luis Obispo, CA) according to manufacturer's instructions. Cell nuclei were counterstained with DAPI (Vector Labs, Burlingame, CA) to assist in quantitation. Four random quadrants of each slide were examined at 20× magnification and the number of apoptotic cells were counted in blinded manner. In addition to TUNEL analysis, the subG1 fraction of cells was measured using flow cytometry to assess apoptosis. Cells were fixed with 70% ethanol overnight at 4°C. Cell pellets were then suspended in 300 µL PBS containing 50 µg/mL propidium iodide (Calbiochem, San Diego, CA) and 100 µg/mL RNase to stain nuclear DNA for 30 min at room temperature. DNA content was analyzed using a Becton Dickinson FACScan flow cytometer (BD Bioscience, San Jose, CA). Quantitation of the subG1 fraction of cells was determined from DNA histograms using BD CellQuest software.

### Matrigel invasion assaya

1×10^5^ cells/mL Panc-1 or MiaPaCa2 cells in serum-free medium was added to the upper chamber of the Boyden chamber coated with Matrigel (coated on a polyvinyl pyrolidone-free polycarbonate filter with 8 µm pore size) inserts (BD Pharmingen, San Diego, CA). The lower chamber contained 10% serum-containing medium. Cells were incubated for 24 hours and invasion of cells to the underside of the Matrigel-coated membrane was detected by staining the cells with Toluidine Blue solution. After staining, cells were counted under a microscope in four random fields in a blinded fashion (magnification 20×).

### Statistical analysis

Statistical analyses were performed using GraphPad Prism software (GraphPad Software, Inc., San Diego, CA). Results are expressed as mean ± S.E.M. Statistically significant differences were determined by Student's *t* test and one-way ANOVA for independent samples where appropriate. p<0.05 was considered statistically significant.
